# Identification and analysis of differentially expressed long non-coding RNAs between multiparous and uniparous goat (*Capra hircus*) ovaries

**DOI:** 10.1371/journal.pone.0183163

**Published:** 2017-09-21

**Authors:** Yinghui Ling, Lina Xu, Long Zhu, Menghua Sui, Qi Zheng, Wenyong Li, Yong Liu, Fugui Fang, Xiaorong Zhang

**Affiliations:** 1 College of Animal Science and Technology, Anhui Agricultural University, Anhui Hefei, China; 2 Local Animal Genetic Resources Conservation and Biobreeding Laboratory of Anhui Province, Anhui Hefei, China; 3 Institute of Plant Protection and Agro-Products Safety, Anhui Academy of Agricultural Sciences, Hefei, Anhui, China; 4 Key Laboratory of Embryo Development and Reproductive Regulation of Anhui Province, Fuyang Normal University, Fuyang, Anhui, China; China Agricultural University, CHINA

## Abstract

Long non-coding RNAs (lncRNAs) play important roles in almost all biological processes. However, there is little information on the effects of lncRNAs on ovulation and lambing rates. In the present study, we used high-throughput RNA sequencing to identify differentially expressed lncRNAs between the ovaries of multiparous (Mul) and uniparous (Uni) Anhui White goats. Among the 107,255,422 clean reads, 183,754 lncRNAs were significantly differentially expressed between the Uni and Mul. Among them, 455 lncRNAs were co-expressed between the two samples, whereas, 157,523 lncRNAs were uniquely expressed in the Uni, and 25,776 uniquely lncRNAs were expressed in the Mul. Through Cis role analysis, 24 lncRNAs were predicted to overlap with cis-regulatory elements, which involved in Progesterone-mediated oocyte maturation, Steroid biosynthesis, Oocyte meiosis, and gonadotropin-releasing hormone (GnRH) signaling pathway. These 4 pathways were related to ovulation, and the KEGG pathway analysis on target genes of the differentially expressed lncRNAs confirmed this results. In addition, 10 lncRNAs harbored precursors of 40 miRNAs, such as TCONS_00320849 related to a mature miRNA sequence, miR-365a, which was reported to be related to proliferation, were annotated in the precursor analysis of miRNAs. The present expand the understanding of lncRNA biology and contribute to the annotation of the goat genome. The study will provide a resource for lncRNA studies of ovulation and lambing.

## Introduction

Long non-coding RNAs (lncRNAs) are non-coding RNA transcripts of more than 200 nucleotides in length. Initially, lncRNA was regarded as transcriptional “noise” that lacked a complete open reading frame and had no ability to encode proteins [1]. Human homologue 19 (H19), found in mice, was the first lncRNA which was discovered in mammals in 1998 [2], then many more lncRNAs were discovered in the following 20 years. Recent studies have shown that lncRNA participates in many important physiological processes, such as X chromosome inactivation [3], dosage compensation [4], and gene imprinting [5]. Additionally, lncRNAs have a significant impact on the diagnosis, treatment, and development of disease. For example, lncRNA plays an important role in epigenetics [6, 7], post-transcriptional regulation [8], maintenance of stem cell pluripotency, and regulation of gene expression during disease processes [9].

Although lncRNAs are widely found in animals, their mechanisms of action are unclear [10, 11]. Using high-throughput poly (A)-independent and strand-specific RNA-seq of rats, lncRNA expression was found to be more tissue specific than that of mRNA [12]. Knockdown of several lncRNAs in mature oocytes increases developmental rates in cattle and leads to larger blastocysts [13]. The sixth CCCTC binding-factor (CTCF) binding site (CCCTC) was identified in H19 DMR, however, had a significant methylation difference between the high- and low-fertility bulls [14]. Boulanger promised that Forkhead box L2 (*Foxl2*) loss of function dissociated from loss of lncRNA expression is sufficient to cause an XX female-to-male sex reversal in the goat model and, as in the mouse model, an agenesis of eyelids [15]. Ovarian hormones regulate *Foxl2* expression and thereby expand the number of genes controlled by the hypothalamic–pituitary–gonad axis that ultimately dictates reproductive fitness [16]. Although lncRNA studies have been begun in several species, the research conducted into the effects of lncRNA on the reproductive rate was relatively limited. High-throughput sequencing can be used to identify the lncRNAs, and bioinformatics analysis can predict the function of lncRNAs, which would provide a basis for animal reproduction study and possibly improve the reproductive rate of goats.

Compared with other goats, Anhui White goat (AWG) is known for its higher fertility, precocious puberty, and higher leather quality. The kidding rate of AWG is 2.27–2.39 and the ewes can be in estrus all year round, which makes it to be an ideal model for the study of goat breeding traits [17]. To explore the function of lncRNAs in ovulation and lambing, we used Solexa sequencing to identify differentially expressed lncRNAs between the ovaries of multiparous and uniparous AWGs in the study, and predict the target genes of the differentially expressed lncRNAs. In addition, classified annotation, GO analysis and KEGG pathway were used to analyze the function of target genes. Through Cis role analysis, 24 lncRNAs were predicted to overlap with cis-regulatory elements, which involved in Progesterone-mediated oocyte maturation, Steroid biosynthesis, Oocyte meiosis, and gonadotropin-releasing hormone (GnRH) signaling pathway. These 4 pathways were related to ovulation, and the KEGG pathway analysis on target genes of the differentially expressed lncRNAs confirmed this results. These results will help us to further understand the role of lncRNAs in ovulation traits.

## Materials and methods

### Experimental animal preparation and library construction

The experimental goats in this study, Anhui White goats (AWG, an indigenous Chinese breed), were obtained from the College of Animal Science and Technology, Anhui Agricultural University, Hefei, China. Based on the long-term observation, laparoscopic and ultrasound detection, 6 target goats, 3 multiparous goats (Mul) and 3 uniparous goats (Uni), which were undergoing oestrus, were selected for their all ovaries. According to the at least 3-year records of lambing, the litter size of Mul and Uni were more than one and only one, respectively. Meanwhile, the physical condition and age, which is 3 to 4 years old, of the experimental samples were basically consistent. To further eliminate the influence of other factors, the unified management system of the field was adopted in the study farm feeding and stabling. The selected goats were killed and dissected, and both whole ovaries from each goat were collected immediately, snap-frozen in liquid nitrogen, and stored at –80°C until use. One whole ovary from each goat was used for RNA-Seq, meanwhile, the other one from the same goat was used for real-time PCR. All experimental procedures involving AWGs performed in the present study had been given prior approval by the ethics committee of Anhui Agricultural University under permit no. AHAU20101025.

The total RNA of the ovaries (12 samples) from the 6 goats was extracted using RNAiso Plus (Takara) following the manufacturer’s protocol. The quantity and quality of the total RNA were measured using the Agilent 2100 Bioanalyzer system. To minimize differences among the goats, an equal quantity (10 μg) of total RNA isolated from one ovary from the 3 individual multiparous and uniparous goats, respectively, were pooled for library preparation and Illumina sequence (The Beijing Genomics Institute). In addition, the total RNA from the other one of the ovaries from the 3 individual multiparous and uniparous goat were reverse transcribed, respectively, into cDNA using the AceQ qPCR SYBR Green Master Mix lncRNA RT-PCR Kit (Vazyme Biotech Co., Ltd.) for qRT-PCR verification.

### Basic data statistics

Before further analysis, raw data filtering was needed to decrease data noise. "Raw reads" were defined as reads containing the adapter sequence, a high content of unknown bases (reads with more than 10% unknown bases), and low-quality reads (>50% low-quality bases in a read, with a low-quality base defined as a base whose sequencing quality was no more than 10). Then clean reads were mapped to the rRNA reference sequence using SOAP aligner/ SOAP2 short-read alignment software [18] to remove the remaining rRNA reads, and leave the reads that were used for transcriptome assembly and quantification. The statistical analysis of the alignment results is presented for each sample.

### Assembly and novel lncRNA prediction

Reads were mapped to the genome and assembled using Cufflinks [19]. Faux-reads were then generated from reference transcripts to capture features in the reference sequence that, due to low coverage, might be missing from the sequencing data. We used a reference annotation-based assembly method to reconstruct the transcripts, while background noise was reduced by using the FPKM (fragments per kilobase of exon per million fragments mapped) value and coverage threshold. These reads were then merged with the sequenced reads for assembly. The set of transcripts generated in the last step was then compared with the reference transcripts to remove the transcripts that were approximately equivalent to the whole or a portion of a reference transcript. Through comparison with the reference, we were able to detect the novel transcripts and calculate the coding potential of these transcripts to identify the novel lncRNA [20]. After the assembly, we compared the assembled transcripts to the reference annotation by Cuffcompare, and categorized the transcripts to 12 classes (=, c, j, e, i, o, p, r, u, x, s and.). Among them, only five candidate categories of transcripts will be extracted, including 'i', 'j', 'o', 'u', and 'x', which may contain novel transcripts. The “u” category meant unknown intergenic transcript. The “x” category meant lncRNAs with exonic overlap with known transcripts, but on the opposite strand. The “i” category contained lncRNAs with transcripts falling entirely within a reference intron. The “j” category meant potentially novel isoforms (fragments) with at least one splice junction shared with the reference transcripts.

### Classification and annotation of lncRNAs

The lncRNAs were also classified by their position compared with the reference gene, with different strategies used for function prediction. For antisense lncRNA, we performed free energy calculation to detect possible hybridization sites for lncRNA and mRNA, which might be RNA-RNA interactions. To further reveal potential antisense lncRNA–mRNA interactions, we searched for all antisense lncRNA-mRNA complementary base pair duplexes using RNAplex [21], a tool specially created to rapidly search for short interactions between two long RNAs. If such interactions are found upstream or downstream of a gene, we will search for lncRNAs located in unknown regions. Because lncRNA can be processed to yield small RNAs, small RNA precursors can be predicted. Recent genome-wide studies suggested that the function of a significant fraction of long non-coding transcripts may be to serve as precursors for microRNAs (miRNAs) that are usually generated via the sequential cleavage of long transcripts by the enzymes Drosha and Dicer [22, 23]. Thus, we aligned lncRNAs to miRBase [24] to detect potential pre-miRNAs by selecting those with a hit coverage greater than 90%.

The SVM-based software miRPara [25] was also used to predict probable miRNAs. In addition, Rfam divides non-coding RNAs into families based on evolution from a common ancestor. To better annotate novel lncRNAs from an evolutionary standpoint, we classified all predicted novel lncRNAs into different non-coding RNA families using INFERNAL, which categorizes non-coding RNAs and their conserved primary sequence and RNA secondary structure through the use of multiple sequence alignments, consensus secondary structure annotation, and covariance models [26]. These results will classify lncRNA families by their RNA structure and sequence similarities, helping us to reveal the potential functions of the lncRNAs.

### Screening of differentially expressed lncRNAs and real-time PCR validation

Cuffdiff was used to calculate expression levels of lncRNAs in more than one condition and test them for significant differences on the basis of FPKM values. To screen differentially expressed genes from the Cuffdiff results, we followed the priority rule: STATUS = OK and P ≤ 0.05. The differentially expressed lncRNAs were screened using NOIseq [27] following the priority rule: filtering condition, log2 (ratio) ≥ 1; probability ≥ 0.8.

Real-time PCR (qPCR) was performed to validate the Solexa sequencing data. Eight differentially expressed lncRNAs between the two libraries were randomly selected for qPCR verification. The primers were designed based on the lncRNA sequences (S1 Table). After incubation at 37°C for 1 h and deactivation at 95°C for 5 min, we obtained the template for qPCR. The reaction solution of qPCR contained 2.0 μl cDNA, 10 μl AceQ qPCR SYBR Green Master Mix and ROX Reference Dye 1, 0.4 μl of each primer, and 6.8 μl ddH_2_O. GAPDH was used as the internal reference gene control. qPCR was performed using standard protocols on the StepOnePlus^™^ Real-Time PCR System (Thermo Fisher Scientific). The threshold cycle (CT) was obtained from each reaction, and the relative expression level of each lncRNA was evaluated using the 2^-ΔΔCt^ method. Three biological replicates for each selected lncRNA were used. The expression levels of lncRNA in the ovaries of Mul and Uni were determined individually and the t-test was used to examine the significant of expression differences between the two samples using SAS v8.0 software.

### Target genes prediction of differentially expressed lncRNAs and function analyses

To explore the functions of lncRNAs, we predicted the target genes of the differentially expressed lncRNAs through Cis role. Cis role is lncRNA action on neighboring target genes. The basic principles of Cis role of target gene is prediction the lncRNA function and the protein coding gene which near the lncRNA’s coordinates. We searched coding genes 100 kb upstream and downstream of lncRNA and then analyzed their function by functional enrichment analysis.

Gene Ontology (GO) and KEGG pathway analyses can help us to understand the biological functions of genes [28]. GO enrichment analysis of lncRNA target genes were implemented by the GOseq R package (http://www.bioconductor.org/packages/release/bioc/html/goseq.html), in which gene length bias was corrected. GO terms with corrected P-value less than 0.05 were considered significantly enriched by differential expressed genes. KEGG is database resource for understand high-level functions and utilities of the biological system, such as the cell, the organism and the ecosystem, from molecular-level information. We used KOBAS software (http://kobas.cbi.pku.edu.cn/download.php) to test the statistical enrichment of lncRNA target genes in KEGG pathways. Pathway with corrected FDR-value less than 0.05 were considered significantly enriched by differential expressed genes.

## Results

### Basic data statistics

In the base composition of the reads, on the X-axis, 1–90 bp represents “read 1” and 91–180 bp represents “read 2”. The A curve should overlap with the T curve, while the G curve should overlap with the C curve. In this case, the figure showed a balanced composition (S1 Fig). By comparing with the rRNA reference sequence through SOAP aligner/ SOAP2 short-read alignment software, 52,178,296 and 55,077,126 clean reads in the Uni and Mul libraries, respectively, were mapped to the genome. Only 0.02% and 0.03% of the total sequence reads were mapped to single-end in the two libraries, respectively. Additionally, only 0.01% of the total sequence reads were mapped to paired-end mapping reads in the two libraries (S2 Table). Otherwise, about 80% of the total mapped reads were mapped to the reference genome, indicating that the quality of the two libraries is good (S3 Table).

### Assembly and novel lncRNA prediction

**Fig 1:**
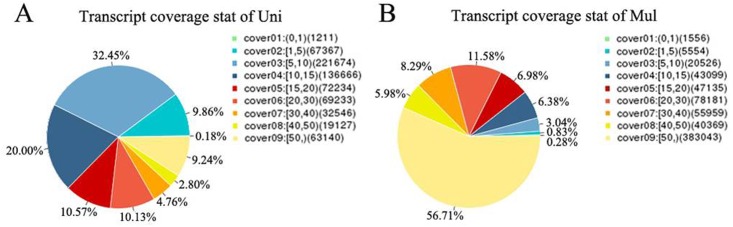
Transcript coverage stat of uniparous goats (Uni) and multiparou goats (Mul).

The clean reads were mapped to the genome using TopHat (Fig 1). There were 663,188 genes were identified in the gene sequencing coverage statistics of the Uni. Among them, transcript coverage on the genome of 63,140 genes was more than50%, which accounted for 9.24% of the identified genes. Meanwhile, 675,422 genes were identified in the Mul library, and 383,043 genes (accounting for 56.71%), whose transcript coverage on the genome was more than 50%. In total, 511,721 lncRNAs and 13,875 mRNAs were identified in the two libraries. Among them, 271,584 lncRNA and 13,224 mRNA were co-expressed in the two libraries, and 240,137 lncRNAs and 651 mRNAs were respectively expressed in the certain library (Table 1).

**Table 1:** Expression statistic of different lncRNAs and mRNA.

Expression type	Novel lncRNA	Known lncRNA	mRNA
**Expressed in 1 sample**	240137	0	651
**Expressed in 2 samples**	271584	2	13224
**Total expressed number(ratio)**	511721	2	13875

Cuffcompare categories were used to distinguish transcripts according to 12 class codes (S4 Table). Only five candidate categories of transcripts will be extracted, including “i”, “j”, “o”, “u” and “x” which may contain novel transcripts. The “u” category contained 525,553 transcripts, which meant unknown intergenic transcript lncRNAs (lincRNAs). The “x” category contained 4,053 transcripts, which meant lncRNAs with exonic overlap with known transcripts, but on the opposite strand. The “i” category contained lncRNAs with transcripts falling entirely within a reference intron. The “j” category meant potentially novel isoforms (fragments) with at least one splice junction shared with the reference transcripts, which contained 38,842 transcripts, and 102 transcripts in “o” category. Due to the complexity of the repeat region, the probability of transcript misassembly is also increased. Thus, we filtered transcripts classified with “r”, which could possibly be artificial fragments. As determined by their genomic locations with respect to nearby genes, the 568,550 lncRNAs included members of all four classes of lncRNAs, with most in the u class, which overlap unknown intergenic transcript lncRNAs. The results of the lncRNA triage were used for further analysis.

### Classification and annotation of lncRNA

By examining the up/downstream lncRNAs of a gene, we found 27,228 lncRNAs had up/downstream gene. Of these, 13,069 lncRNAs were located in the antisense strand and 14,159 were located in the sense strand. Therefore, 49% of lncRNAs were downstream of the bracketing genes, and the others were upstream. These lncRNAs could overlap with cis-regulatory elements probably involved in transcriptional regulation. TCONS_00076241, TCONS_00087806, and TCONS_00076240 were upstream of XM_005686239.1 (BUB1) in the oocyte meiosis pathway. TCONS_00248477 was downstream of XM_005676195.1 (MAP3K19) in the GnRH signaling pathway. TCONS_00136407 and TCONS_00146968 were downstream of XM_005689083.1 (MOS) in the progesterone-mediated oocyte maturation pathway. TCONS_00136407 and TCONS_00146968 were downstream of XM_005689083.1 (MOS) in the oocyte meiosis pathway. MOS, BUB1, and MAP3K19 may regulate oocyte meiosis (Table 2).

**Table 2:** Up/down stream lncRNA of a gene.

Pathway	lncRNA ID	Chr	strand	Braketing gene	Up/down stream
**Progesterone-mediated oocyte maturation**	TCONS_00390275	chr3	-	XM_005678520.1	UPSTREAM_2K
	TCONS_00136407	chr14	+	XM_005689083.1	DOWNSTREAM_2K
	TCONS_00146968	chr14	-	XM_005689083.1	DOWNSTREAM_2K
**Steroid biosynthesis**	TCONS_00376376	chr3	+	XM_005678362.1	DOWNSTREAM_2K
	TCONS_00376377	chr3	+	XM_005678362.1	DOWNSTREAM_2K
**Oocyte meiosis**	TCONS_00386107	chr3	-	XM_005678170.1	UPSTREAM_2K
	TCONS_00386108	chr3	-	XM_005678170.1	UPSTREAM_2K
	TCONS_00094458	chr11	-	XM_005686832.1	UPSTREAM_2K
	TCONS_00094457	chr11	-	XM_005686832.1	UPSTREAM_2K
	TCONS_00259604	chr20	+	XM_005694802.1	UPSTREAM_2K
	TCONS_00267219	chr20	-	XM_005694802.1	DOWNSTREAM_2K
	TCONS_00071211	chr10	-	XM_005685706.1	DOWNSTREAM_2K
	TCONS_00071210	chr10	-	XM_005685706.1	DOWNSTREAM_2K
	TCONS_00061051	chr10	+	XM_005685706.1	DOWNSTREAM_2K
	TCONS_00136407	chr14	+	XM_005689083.1	DOWNSTREAM_2K
	TCONS_00146968	chr14	-	XM_005689083.1	DOWNSTREAM_2K
	TCONS_00076241	chr11	+	XM_005686239.1	UPSTREAM_2K
	TCONS_00087806	chr11	-	XM_005686239.1	UPSTREAM_2K
	TCONS_00076240	chr11	+	XM_005686239.1	UPSTREAM_2K
	TCONS_00322561	chr25	+	XM_005697767.1	UPSTREAM_2K
	TCONS_00322565	chr25	+	XM_005697767.1	DOWNSTREAM_2K
	TCONS_00322562	chr25	+	XM_005697767.1	UPSTREAM_2K
**GnRH signaling pathway**	TCONS_00248477	chr2	-	XM_005676195.1	DOWNSTREAM_2K
	TCONS_00386107	chr3	-	XM_005678170.1	UPSTREAM_2K

Note: Pathway, the KEGG pathway; lncRNA ID, the ID of lncRNA; Chr: chromosome which is the lncRNA came from; Strand, the strand which the lncRNA located; Braketing gene, the nearest gene in upstream or downstream; Up/down stream, whether upstream or downstream is the lncRNA located to a gene.

**Fig 2:**
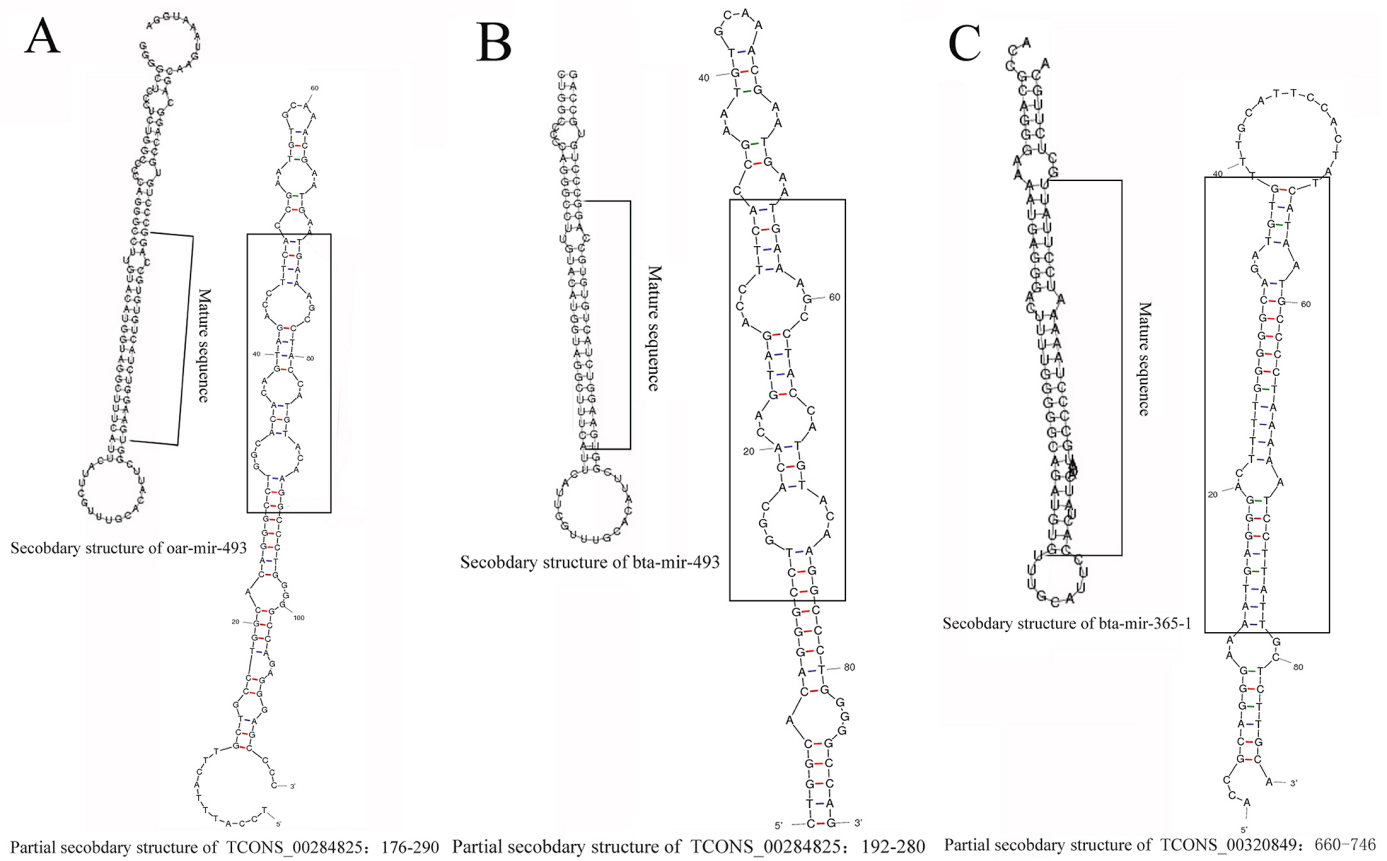
The pre-miRNA and secondary structure of oar-mir-493, bta-mir-493 and bta-mir-365-1. Note: A, known pre-miRNA and secondary structure pictures of oar-mir-493; B, known pre-miRNA and secondary structure pictures of bta-mir-493; C, known pre-miRNA and secondary structure pictures of bta-mir-365-1.

We selected 65,536 lncRNAs which coverage were greater than 90% to examine the regulatory functions of the lncRNAs. We aligned lncRNAs to miRBase to determine if they could target known miRNAs: TCONS_00320849 had 16 known precursors, TCONS_00284825 had 10 known precursors, TCONS_00080255 had 7 known precursors, TCONS_00284070, TCONS_00304715, TCONS_00424188, TCONS_00533458, TCONS_00308106, TCONS_00087926, and TCONS_00205426 had 1 known precursor, respectively. Bta-mir-365-1, cfa-mir-493, and bta-mir-493 are precursors of TCONS_00284825, whereas oar-mir-493 is the precursor of TCONS_00320849, which the identities were 100%. We found that TCONS_00087926, TCONS_00304715, TCONS_00424188, and TCONS_00320849 had several novel precursors, but TCONS_00320849 was related to a mature miRNA sequence, miR-365a, which had the same mature miRNA in the miRBase database (Fig 2, S5 and S6 Tables).

To better annotate the novel lncRNA from an evolutionary standpoint, we classified all of the predicted novel lncRNAs into different non-coding RNA families using INFERNAL. Fifty families were obtained via the lncRNA family prediction. The C/G content was between 25% and 70%. Xist_exon1, TUG1_3, TUG1_4, SOX2OT_exon3, and SMAD5 may regulate reproduction and embryonic development [29–31] (Table 3).

**Table 3:** Families prediction of lncRNAs.

Family Name	Family Accession	LncRNA	Start	End	E-value	Score	GC%
**MALAT1**	RF01871	TCONS_00360963	3768	3859	3.70E-08	58.2	0.25
**MIAT_exon5_2**	RF01876	TCONS_00194619	616	700	1.20E-20	99.5	0.54
**MIAT_exon5_2**	RF01876	TCONS_00202016	318	234	1.20E-20	99.5	0.54
**MIAT_exon5_3**	RF01877	TCONS_00194620	170	427	7.30E-82	282.5	0.6
**MIAT_exon5_3**	RF01877	TCONS_00194621	1	62	8.70E-17	68.9	0.48
**Xist_exon1**	RF01880	TCONS_00543946	2128	2044	2.60E-24	102.8	0.52
**BC040587**	RF01884	TCONS_00164431	407	269	0.00064	35.5	0.47
**Evf1_1**	RF01887	TCONS_00393382	205	347	2.70E-26	133.4	0.31
**TUG1_3**	RF01891	TCONS_00194967	988	1229	8.40E-69	232.6	0.5
**TUG1_4**	RF01892	TCONS_00194967	1536	1712	5.00E-51	195.8	0.4
**CDKN2B**	RF01909	TCONS_00190200	432	580	0.0062	29.3	0.36
**KCNQ1OT1_2**	RF01947	TCONS_00500370	299	141	0.00042	26.5	0.65
**KCNQ1OT1_2**	RF01947	TCONS_00512589	562	720	0.00042	26.5	0.65
**KCNQ1OT1_2**	RF01947	TCONS_00081060	183	56	0.0098	22.3	0.52
**SOX2OT_exon3**	RF01953	TCONS_00031909	362	87	1.20E-101	321.8	0.51
**SOX2OT_exon3**	RF01953	TCONS_00048514	14	289	1.20E-101	321.8	0.51
**KCNQ1DN**	RF01961	TCONS_00329408	230	170	0.005	35.7	0.28
**RMST_2**	RF01963	TCONS_00423556	43	183	5.10E-28	125.3	0.5
**RMST_7**	RF01968	TCONS_00423569	62	328	1.50E-92	310	0.34
**RMST_10**	RF01971	TCONS_00423578	239	388	1.50E-26	110.4	0.51
**ZEB2_AS1_1**	RF01984	TCONS_00247591	235	361	5.30E-36	147.2	0.49
**ZEB2_AS1_1**	RF01984	TCONS_00232918	373	247	5.30E-36	147.2	0.49
**ZEB2_AS1_1**	RF01984	TCONS_00274957	4175	4300	0.0056	29	0.4
**ZEB2_AS1_2**	RF01985	TCONS_00232917	826	766	4.70E-16	86.6	0.43
**DAOA**	RF02091	TCONS_00115217	373	174	4.10E-53	183.6	0.49
**DLEU1_1**	RF02103	TCONS_00100306	7	197	1.30E-49	187.6	0.48
**DLG2**	RF02112	TCONS_00357136	2081	1893	6.30E-46	195.5	0.39
**DLG2**	RF02112	TCONS_00168067	201	392	1.20E-28	128.7	0.28
**DLG2**	RF02113	TCONS_00357133	405	216	3.70E-39	139.3	0.55
**SMAD5**	RF02174	TCONS_00052610	280	178	0.0028	27.4	0.48
**SMAD5**	RF02174	TCONS_00036078	362	464	0.0028	27.4	0.48
**ST7**	RF02179	TCONS_00410144	419	479	3.70E-06	48.9	0.34
**ST7**	RF02187	TCONS_00410143	198	391	3.40E-42	194.9	0.41
**ST7**	RF02188	TCONS_00410143	672	857	1.10E-30	135.9	0.42
**ST7**	RF02189	TCONS_00410142	155	323	2.60E-21	82.4	0.44
**TCL6_1**	RF02191	TCONS_00276890	1	78	1.70E-07	51	0.5
**TTC28**	RF02199	TCONS_00126228	291	431	0.0062	36	0.37
**TTC28**	RF02200	TCONS_00148497	935	766	0.0022	25.8	0.48
**WT1**	RF02204	TCONS_00159836	411	539	2.20E-14	77.6	0.55
**WT1**	RF02205	TCONS_00159836	909	1143	3.30E-53	184	0.65
**WT1**	RF02206	TCONS_00159836	1186	1307	2.80E-15	82.6	0.59
**WT1**	RF02208	TCONS_00159836	3187	3308	3.60E-24	115	0.7
**WT1**	RF02209	TCONS_00159837	342	635	2.50E-106	337.5	0.47
**WT1**	RF02210	TCONS_00159837	733	1003	1.10E-68	264.5	0.31
**ZNFX1**	RF02215	TCONS_00123796	1	59	7.70E-08	42.4	0.68
**ZNFX1**	RF02216	TCONS_00123796	179	270	7.50E-11	68.8	0.59
**Six3os1_2**	RF02247	TCONS_00114628	100	283	0.0028	24.9	0.47
**Six3os1_2**	RF02247	TCONS_00349364	424	247	0.007	23.6	0.48
**adapt33_2**	RF02256	TCONS_00462772	583	512	0.00031	39.2	0.4
**adapt33_2**	RF02256	TCONS_00462773	583	512	0.00031	39.2	0.4

Note: Family Name, assigned Family name in Rfam database; Family Accession, Correspond Family accession number; LncRNA, long noncoding RNA; Start, start site of the alignment; End, end site of the alignment; E-value, expect value for the alignment; Score, score of the alignment given by Infernal; GC%, GC content of the conserved region; Rank, Rank of this lncRNA in the corresponding family.

### Differentially expressed lncRNAs and real-time PCR Validation

**Fig 3:**
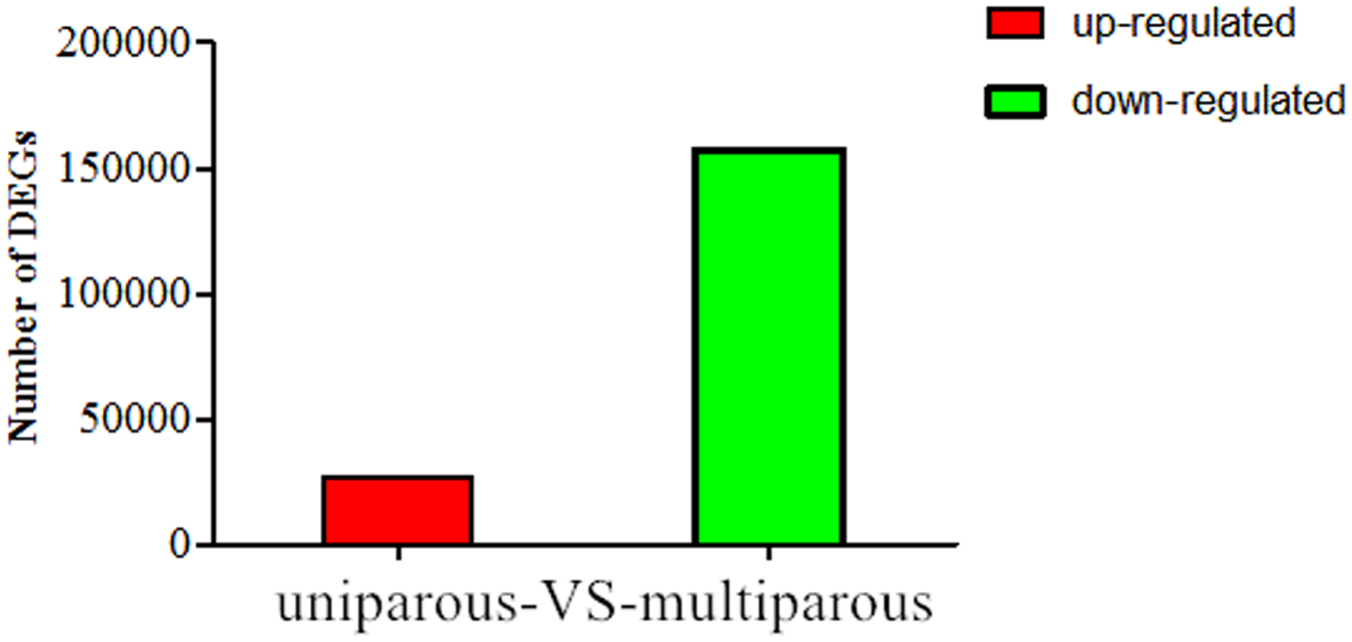
Stat chart of up-down-regulated expressed genes.

Compared to the Uni library, 26,187 up-regulated and 157,567 down-regulated lncRNAs were identified in the Mul library (Fig 3). A total of 183,754 lncRNAs were significantly differently expressed between the Mul and Uni with |log2Ratio|2 ≥ 1 and p-value ≤ 0.05. Among them, 455 lncRNAs, which included 188 known genes and 267 predicted transcripts, were co-expressed in the two samples. In the Mul, the highest expressed lncRNA from the co-expressed lnRNAs was XLOC-013414, which FPKM value was 5052.08, whereas, its FPKM in the Uni was 572.03. In the Uni, the highest expressed lncRNA from the co-expressed lnRNAs was 102188530, which FPKM value was 741.67, whereas, its FPKM in the Mul was 109.21. In addition, 157,523 lncRNAs were uniquely expressed in the Uni, and 25,776 uniquely lncRNAs were expressed in the Mul. The expression of uniquely expressed lncRNA was relatively low. Such as the expression of XLOC-505751, which was highest uniquely expressed in the Uni, was 60.29. And the expression of XLOC-263636, which was highest uniquely expressed in the Mul, was 70.38 (S7 Table).

**Fig 4:**
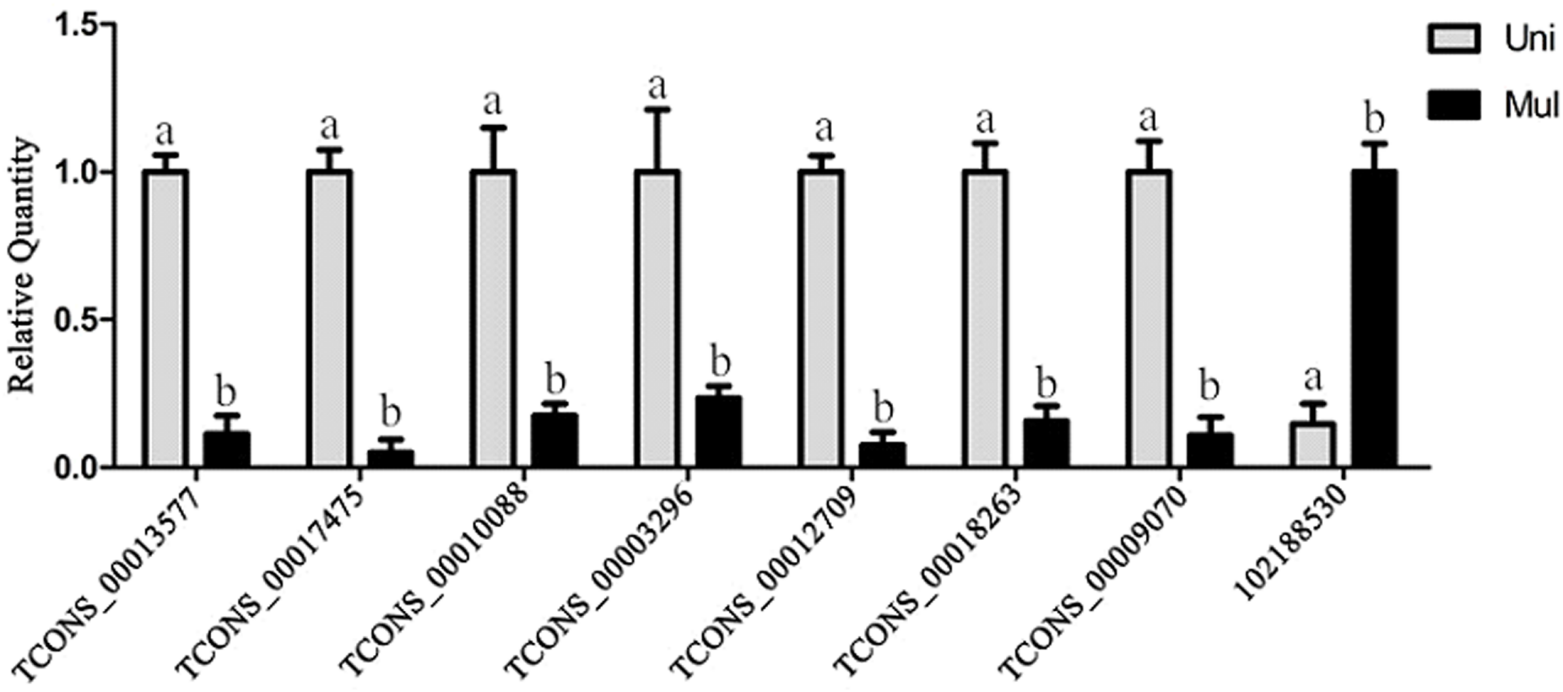
Real-time PCR results of randomly selected differentially expressed lncRNAs. Note: X-axis represents selected 8 differential expressed transcripts in two libraries. Here, GAPDH was chosen as the reference gene. Relative expression value per selected transcripts between uniparous goats (Uni) and multiparous goats (Mul) samples was calculated (y-axis). Superscript letters indicate significant difference at the level of 0.05.

To ensure the accuracy and reliability of the RNA-seq results, eight differentially expressed lncRNAs were randomly selected for qPCR to confirm the expression changes. The results showed good consistency between the RNA-seq results and the qPCR results (Fig 4).

### Function analyses of target genes of differentially expressed lncRNAs

The target genes of the differentially expressed lncRNAs were predicted through Cis role. After searching the coding genes 100 kb upstream and downstream of lncRNA, we analyzed the function of the target genes through GO and KEGG enrichment analysis. In total, there were 32,627 genes were predicted as the target genes of differentially lncRNAs between the Uni and Mul (S8 and S9 Tables).

Based on the gene background, there were 439, 432, and 407 target genes clustered into 141, 198, and 868 GO terms in component, function and process ontology, respectively. The GO terms “Cell part”, “Binding” and “Cellular process” were the biggest terms, which included the most target genes, in the above ontologies, respectively. However, compared with the reference gene background, no GO term was significantly enriched (p-value ≤ 0.05). The “Phosphatidylinositol binding” and “Cyclic-nucleotide phosphodiesterase activity” in function ontology, and “Cholesterol metabolic process”, “Cell aging”, “Aging”, “Steroid metabolic process” in process ontology were the only GO terms with corrected p-value < 1 (S10 Table).

According to the KEGG pathway analysis, a total of 875 target genes, which showed 22,885 background genes, of the differentially expressed lncRNAs between the Uni and Mul, were annotated for 236 pathways. Among them, 12 pathways, “mTOR signaling pathway (ko04150)”, “Measles (ko05162)”, “Olfactory transduction (ko04740)”, “Regulation of autophagy (ko04140)”, “Insulin signaling pathway (ko04910)”, “RNA degradation (ko03018)”, “RNA polymerase (ko03020)”, “Nucleotide excision repair (ko03420)”, “Adipocytokine signaling pathway (ko04920)”, “Fatty acid biosynthesis (ko00061)” “Purine metabolism (ko00230)”, and “SNARE interactions in vesicular transport (ko04130)”, were significantly enriched (p-value ≤ 0.05). In addition, five pathways, “Progesterone-mediated oocyte maturation (ko04914) (p-value = 0.1484)”, “Steroid biosynthesis (ko00100) (p-value = 0.1511)”, “Oocyte meiosis (ko04114) (p-value = 0.1963)”, “GnRH signaling pathway (ko04912) (p-value = 0.5711)”, and “Steroid hormone biosynthesis (ko00140) (p-value = 0.2323)” were related to the animal reproduction [32–34] (S11 Table).

## Discussion

In the study, we used RNA-seq to study the lncRNAs expressed in the ovaries of multiparous (Mul) and uniparous (Uni) goat to explore the function of lncRNAs in ovulation and lambing. In total, 107,255,422 clean reads were obtained from the two libraries, and 52,178,296 and 55,077,126 clean reads in the Uni and Mul libraries, respectively, were mapped to the genome. However, a small part of the lncRNA sequence still could not be aligned to the target genome and located to a specific chromosome. Because the sequence was broken up into smaller pieces called segments, with the software inferring that the read spans a splice junction and estimating the location of the splice sites of the junction. On one hand, we believed that this shortcoming may be because the reference goat genome is still not perfect, with more pieces to be spliced. On the other hand, there may be post-transcriptional RNA editing, resulting in inconsistencies between the lncRNA sequence and the corresponding DNA sequence. Some lncRNAs highly expressed in the ovary may lack a classical poly (A) tail [35]. In the renal medulla, 28% of lncRNAs were found to lack a classical poly (A) tail [12]. Strand-specific sequences rather than 3’-directed cDNA sequences were used in this study. Strand-specific sequences help to determine the polarity of transcripts and improve the accuracy of the quantification of antisense transcripts. When lncRNA and mRNA were isolated from total RNA, the ribo-depleted method was used instead of the poly (A) enrichment method. Large-scale bioinformatic studies suggest that a significant fraction (>24%) of long non-coding transcripts present in cells may lack a classical poly (A) tail.

In this study, we searched for lncRNAs located in unknown regions if they could be found upstream or downstream of a gene to predict the function of the lncRNA. TCONS_00076241, TCONS_00087806, and TCONS_00076240 were upstream of XM_005686239.1 (BUB1). TCONS_00248477 was downstream of XM_005676195.1 (MAP3K19). TCONS_00136407 and TCONS_00146968 were downstream of XM_005689083.1 (MOS). Studies have shown that XM_005689083.1 and XM_005686239.1 play a role in meiosis I in the oocyte [36]. XM_005676195.1 may regulate GnRH in the GnRH signaling pathway. Bta-mir-365-1, cfa-mir-493 [37], and bta-mir-493 [38] are precursors of TCONS_00284825 and oar-mir-493 [39] is a precursor of TCONS_00320849 in pre-miRNA prediction. TCONS_00320849 has a mature miRNA sequence, miR-365a, with the same mature sequence in the miRBase database. miR-365 can regulate proliferation by mediating the expression of KRAS [40]. miR-365 regulates cell cycle progression and apoptosis in various types of tumors [41]. Thus TCONS_00136407, TCONS_00146968, and TCONS_00320849 may also play roles in meiosis in the oocyte.

To further detect the lncRNAs, which may participate in the ovulation and lambing of goat, we analyzed the differentially expressed lncRNAs between the Uni and Mul. A total of 183,754 lncRNAs were significantly differently expressed between the two libraries. Among them, 455 lncRNAs, which included 188 known genes and 267 predicted transcripts, were co-expressed between the two samples. In addition, 157,523 lncRNAs were uniquely expressed in the Uni, and 25,776 lncRNAs were uniquely expressed in the Mul. Accordingly, we analyzed the lncRNAs which have high expression levels and were significantly difference expressions between the Uni and Mul libearies. XLOC_013414 was differentially expressed lncRNA with the highest expression level in the Uni library, whereas 102188530 (COL1A1) had the highest expression in the Mul library. The function of XLOC_013414 is unknown but COL1A1 polymorphisms may individually play minor roles in osteoporosis and fracture [42]. Meanwhile, XLOC_500835, XLOC_075014, XLOC_523784 were significantly differentially expressed novel lncRNA between the Uni and the Mul. And their target genes were KIAA0368, CNGA3 and MID2, respectively. KIAA0368 as selectively up-regulated by contact allergene, may be potential markers of skin irritation and allergy [43]. Achromatopsia can be caused by mutations in the CNGA3 gene [44]. MID2 leads to misregulation of microtubule organization and the downstream disease pathology associated with X-linked intellectual disabilities [45]. In consideration of the specific and higher expression levels of XLOC_013414 and COL1A1, some further research of their roles in the ovary is warranted.

According to the KEGG pathway analysis, a total of 875 target genes, which showed 22,885 background genes, of the differentially expressed lncRNAs between the Uni and Mul were annotated to 236 pathways. Among them, 12 pathways were significantly enriched (p-value ≤ 0.05). In addition, five pathways, “Progesterone-mediated oocyte maturation”, “Steroid biosynthesis”, “Oocyte meiosis”, “GnRH signaling pathway”, and “Steroid hormone biosynthesis” were related to the animal reproduction. An increased FSH secretion level will lead to multiple follicular development and ovulation in the follicular phase [46–48]. In addition, Neat1 knockout mice fail to become pregnant despite normal ovulation [49]. Ovarian hormones can regulate the expression of Foxl2 to expand the number of genes controlled by the hypothalamic–pituitary–gonadal axis, ultimately dictating reproductive fitness [16].

In summary, the differentially expressed profile of lncRNAs was successfully established in the Uni and Mul phase libraries. Through Cis role analysis, 24 lncRNAs were predicted to overlap with cis-regulatory elements, which involved in Progesterone-mediated oocyte maturation, Steroid biosynthesis, Oocyte meiosis, and gonadotropin-releasing hormone (GnRH) signaling pathway. Meanwhile, the KEGG pathway analysis on target genes of the differentially expressed lncRNAs confirmed this results. In addition, 10 lncRNAs harbored precursors of 40 miRNAs, which was reported to be related to proliferation, were annotated in the precursor analysis of miRNAs. The present expand the understanding of lncRNA biology and will provide a resource for lncRNA studies of ovulation and lambing.

## Supporting information

S1 Fig Base composition analysis of sample Uni and Mul. (JPG)

S1 Table Summary of lncRNA primers sequences for the RT-PCR. (XLSX)

S2 Table Statistics of clean data mapping to rRNA by soap. (XLSX)

S3 Table Statistics of clean data without rRNA mapping to genome by tophat. (XLSX)

S4 Table Cuffcompare category to distinguish transcripts by 12 symbols. (XLS)

S5 Table Result of comparison with known pre-miRNA. (XLS)

S6 Table Result of pre-miRNA prediction. (XLSX)

S7 Table The differentially expressed lncRNAs between the two libraries. (XLSX)

S8 Table The protein-coding genes within 100 k downstream of an lncRNA. (XLSX)

S9 Table The protein-coding genes within 100 k upstream of an lncRNA. (XLSX)

S10 Table 10 GO analysis for the target genes of the differentially expressed lncRNAs. (XLSX)

S11 Table KEGG pathways for the target genes of the differentially expressed lncRNAs. (XLS)
