# Supplementary figures and images for: Identification and analysis of differentially expressed long non-coding RNAs between multiparous and uniparous goat (Capra hircus) ovaries
Source: PLoS One. 2017 Sep 21;12(9):e0183163. doi: 10.1371/journal.pone.0183163 (PMC5608193; doi:10.1371/journal.pone.0183163)

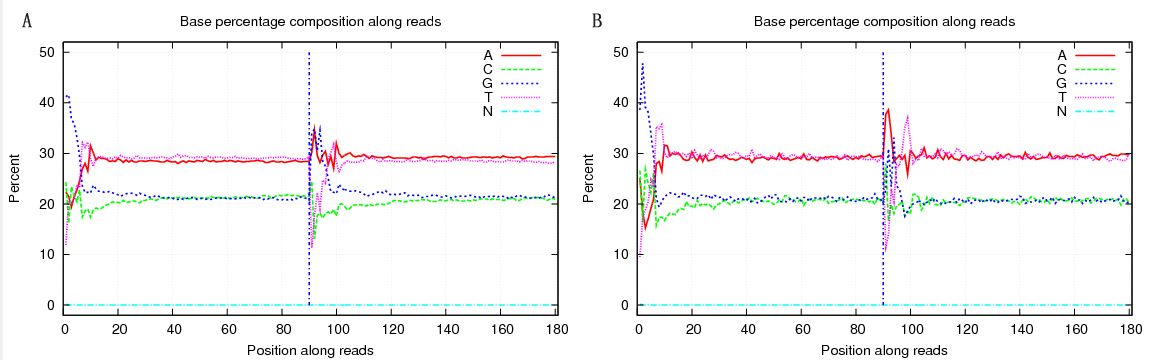

Supplement: S1 Fig — (JPG) [file pone.0183163.s001.jpg]
